# Overexpression of EIF5A2 Predicts Poor Prognosis in Patients with Oral Squamous Cell Carcinoma

**DOI:** 10.3390/diagnostics10070436

**Published:** 2020-06-27

**Authors:** Yueh-Min Lin, Mei-Ling Chen, Chia-Lo Chen, Chung-Min Yeh, Wen-Wei Sung

**Affiliations:** 1 School of Medicine, Chung Shan Medical University, Taichung 40201, Taiwan; 93668@cch.org.tw (Y.-M.L.); 55828@cch.org.tw (M.-L.C.); dodopenguin24@gmail.com (C.-L.C.); 2 Department of Surgical Pathology, Changhua Christian Hospital, Changhua 50006, Taiwan; 28935@cch.org.tw; 3 Department of Medical Technology, Jen-Teh Junior College of Medicine, Nursing and Management, Miaoli 35664, Taiwan; 4 Institute of Medicine, Chung Shan Medical University, Taichung 40201, Taiwan; 5 Department of Urology, Chung Shan Medical University Hospital, Taichung 40201, Taiwan

**Keywords:** eukaryotic translation initiation factor 5A2, EIF5A2, prognosis, oral cancer, oral squamous cell carcinoma, overall survival, epithelial–mesenchymal transition

## Abstract

Oral squamous cell carcinoma (OSCC) is the most common epithelial malignancy affecting the oral cavity, and it is especially significant in Asian countries. Patients diagnosed with OSCC have an unfavorable prognosis and additional prognostic markers would help improve therapeutic strategies. We sought to investigate the association between eukaryotic translation initiation factor 5A2 (EIF5A2) and epithelial–mesenchymal transition (EMT) markers as well as the prognostic significance of EIF5A2 in OSCC. The expression of EIF5A2 and EMT markers was measured through the immunohistochemical staining of specimens from 272 patients with OSCC. In addition, the correlation between different clinicopathological factors and EIF5A2 expression was analyzed. The prognostic role of EIF5A2 was then analyzed via Kaplan–Meier analysis and Cox proportional hazard models. Among the 272 patients, high EIF5A2 expression was significantly associated with an advanced N value (*p* = 0.008). High tumor expression of EIF5A2 was prone to the expression of low E-cadherin and high beta-catenin (*p* = 0.046 and *p* = 0.020, respectively). Patients with high EIF5A2 expression had unfavorable five-year survival rates as compared with those with low expression (49.7% and 67.3%, respectively). The prognostic role of EIF5A2 was further confirmed through multivariate analysis (hazard ratio = 1.714, 95% confidence interval: 1.134–2.590, *p* = 0.011). High EIF5A2 expression is associated with an advanced N value and EMT markers and may serve as a marker for an unfavorable prognosis in patients with OSCC.

## 1. Introduction

Oral cancer is the sixth most common cancer among men worldwide, and the number of new cases increases every year [1]. Incidence rates not only vary by gender and age but also show geographical differences. In addition to well-known risk factors such as tobacco, alcohol, poor nutrition, and industrial pollution, betel quid chewing may be a possible reason for why South and Southeast Asian countries are characterized by a high incidence rate (12.7 per 100,000) [2,3]. In our population in Taiwan, the incidence rate for males has risen to 29.2 per 100,000 annually, indicating that oral cancer is a major issue in this area [3]. According to the American Society of Clinical Oncology, the five-year survival rate for localized cancer is around 84%, while distant oral cancer reduces the rate to 39% [1]. Patients at N2 and N3 stages have a poor prognosis and high rate of recurrence even after standard treatment [4,5]. As a result, exploring prognostic markers that can predict potential advanced stage oral cancer patients for the purpose of constructive monitoring and treatment is important.

In regard to the complexity of the carcinogenesis process interfering with genotype and phenotype levels, factors associated with cell proliferation, cell apoptosis, growth inhibition, and more have been discovered as prognostic biomarkers [6]. Over the decades, hundreds of biomarkers have been investigated, including p53, Ki-67, p16, Cyclin D1, and VEGFs, with variable conclusions [7]. The eukaryotic translation initiation factor 5A2 (EIF5A2) expressing gene is located on chromosome 3q26, which is a location for many other oncogenes [8,9,10]. As a member of the EIF5A family, which promotes the initiation of mRNA translation, EIF5A2 is also involved in cell proliferation, apoptosis, and nuclear export [11,12]. Unlike most members of the EIF5A family that are universally expressed, EIF5A2 is tissue specific to the testis, brain, and tumor cells, especially in ovarian and colorectal cancer cell lines [9,13]. EIF5A2 has been reported to be associated with ovarian cancer, colorectal carcinoma, hepatocellular carcinoma, breast cancer, bladder cancer, non-small cell lung cancer, gastric cancer, prostate cancer, and nasopharyngeal carcinoma [8,9,14,15,16,17,18,19,20].

Studies of the molecular mechanism of EIF5A2 related to carcinogenesis indicate that the overexpression of EIF5A2 induces epithelial–mesenchymal transition (EMT). In hepatocellular carcinoma, the overexpression of EIF5A2 activates the RhoA/Rac1 signaling pathway to stimulate cytoskeleton rearrangement [20]. EIF5A2 overexpression in colorectal carcinoma induces EMT by upregulating MAT-1 through c-Myc [21]. The induction of EMT in gastric cancer cells has also been proven to have a relationship with EIF5A2 overexpression while it decreases the cell sensitivity of cisplatin [10].

Although previous research suggests EIF5A2 as a possible biomarker and therapeutic target in several cancers, the relation between oral squamous cell carcinoma (OSCC) and EIF5A2 expression remains unknown. Therefore, this study evaluated the expression and clinical significance of EIF5A2 in OSCC tumors and its correlation with EMT markers. We also investigated the prognostic role of EIF5A2 in OSCC patients according to their clinicopathological parameters.

## 2. Materials and Methods

### 2.1. Patients

Our study examined 272 non-metastatic tumor samples from patients with surgical intervention for OSCC in oral cavity between 2000 and 2007. Patients were staged according to the seventh edition of American Joint Committee on Cancer Cancer Staging Manual. The clinicopathological features collected included risk factors, histological type, differentiation, and TNM (Tumor, Node, Metastasis) stage taken from the established database [22,23]. The histological diagnoses had been previously confirmed by two pathologists [22,23]. Patients with primary OSCC and who had surgical resection were included. Those patients with a previous cancer history who were missing clinical data were excluded from this study to reduce bias from missing data. The study was approved by the Institutional Review Board and Ethics Committee of Changhua Christian Hospital, Changhua, Taiwan (IRB No. 180713, 1 February 2019).

### 2.2. Immunohistochemical Staining

The immunohistochemical (IHC) staining was performed at the Department of Surgical Pathology, Changhua Christian Hospital, using anti-human EIF5A2 antibody (Sigma E9781; 1:75 dilution), anti-human beta-catenin (Cell Marque (14); 1:75 dilution), and E-cadherin (Bio SB SBS5467; 1:100 dilution) as previously described [24]. There are 10 and 4 missing data tissue detachment during IHC staining in E-cadherin and beta-catenin. Immunoreactivity scores were analyzed by the pathologists using a previously described scoring protocol [24,25]. The immunoreactivity scores were defined as cell staining intensity (0–3) multiplied by the percentage of stained cells (0%–100%), leading to scores from 0 to 300 [24,25,26]. Staining of proteins was assessed semiquantitatively by 2 pathologists, who scored coded sections independently based on the staining score and were blind to the prognostic data of the study. A final agreement was obtained for each score, even for discrepant immunostaining results. A final agreement was obtained for each score by using a multiheaded microscope (Olympus BX51 10 headed microscopes).

### 2.3. Patient and Public Involvement

This study analyzed cancer tissues from a delinked database. Therefore, we did not inform or disseminate information to the patients regarding the research project, outcome investigation, or results. The patients were not involved in the study, including not being involved in the design, recruitment, or conduct of the study. There was no patient adviser for the contributorship statement.

### 2.4. Statistical Analyses

A χ^2^ test was applied for the continuous and discrete data analysis. The associations between EIF5A2 expression and overall patient survival were estimated using univariate analysis and the Kaplan–Meier method and assessed further using the log-rank test [25,27]. Potential confounders, including age, gender, smoking, betel quid chewing, and stage, were adjusted for using Cox regression models of multivariate analysis, with EIF5A2 expression fitted as an indicator variable. All statistical analyses were conducted using SPSS statistical software (version 15.0; SPSS Inc., Chicago, IL). All statistical tests were two-sided, and values of *p* < 0.05 were considered statistically significant.

## 3. Results

### 3.1. Relationships Between EIF5A2 Expression and Clinical Parameters in Oral Cancer Patients

**Figure 1:**
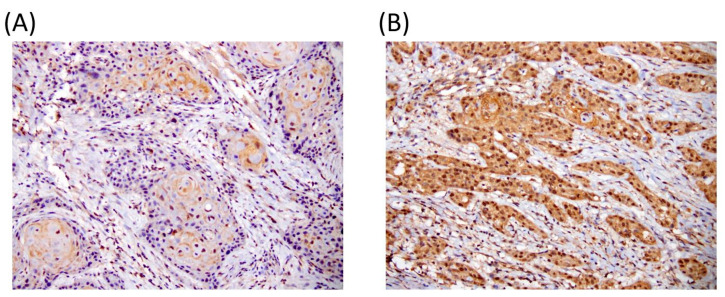
Representative immunostaining of (**A**) low and (**B**) high EIF5A2 expression of oral squamous cell carcinoma (OSCC) specimens.

The expression of EIF5A2 was investigated by IHC using tissue of primary tumor including 272 oral cancer patients (Figure 1).

Expression of EIF5A2 was divided into two levels according to the percentage and intensity of the IHC staining and the cut-off score was 80. For all of the 272 patients, the EIF5A2 expression level was not significantly associated with age, gender, smoking, betel quid chewing, or staging (Table 1). Notably, patients with regional lymph node metastasis tended to have higher tumor EIF5A2 expression (73.1%, *p* = 0.008; Table 1).

### 3.2. EIF5A2 Expression of is Associated with EMT Markers in Oral Cancer Specimens

In order to determine the EMT markers among different levels of EIF5A2 expression in the oral cancer patients, we further detected E-cadherin and beta-catenin protein expression. There are 10 and 4 with missing data due to tissue detachment during IHC staining of E-cadherin and beta-catenin. Patients with higher EIF5A2 expression showed a tendency toward lower levels of E-cadherin (71.1% vs.59.9%, *p* = 0.046; Table 2) and higher levels of beta-catenin (57.0% vs 70.7%, *p* = 0.020; Table 2).

### 3.3. EIF5A2 is an Independent Factor Associated with Oral Cancer Overall Survival

We performed univariate and multivariate analyses of various parameters. The mean survival time was 4.6 years, the median survival time was not reached, and the median follow-up time was 3.7 years. In the Cox regression model, the selected explanatory variables were age, gender, smoking, betel quid chewing, stage, and EIF5A2 expression. The five-year survival rate for stage Ⅰ patients was 74.4% as compared to 57.9% for stage Ⅱ to Ⅳ patients (HR = 1.762, 95% confidence interval [CI]: 1.082–2.868, *p* = 0.023; Table 3). The five-year survival rate for N0 patients was 68.4% as compared to 36.8% for N1 to N3 patients (HR = 2.770, 95% CI: 1.906–4.024, *p* < 0.001; Table 3).

**Figure 2:**
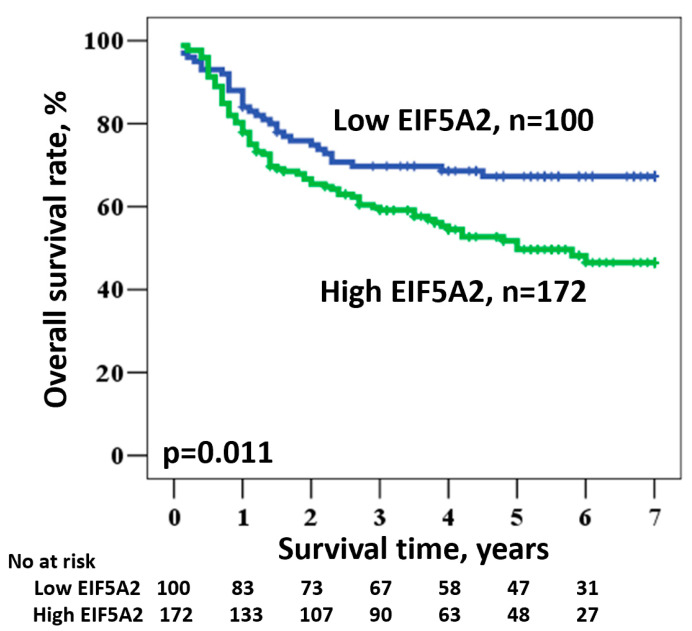
Kaplan–Meier survival of OSCC patients according to EIF5A2 expression.

Expression level of EIF5A2 is associated with an even worse five-year survival rate of 49.7% for high and 67.3% for low expression levels (HR = 1.696, 95% CI: 1.126–2.554, *p* = 0.011; Figure 2 and Table 3).

A multivariate analysis was used to clarify the independent prognostic role of EIF5A2 expression in oral cancer patients. After adjusting for age, gender, smoking, betel quid chewing, and stage, EIF5A2 expression remained statistically significant with the overall survival of oral cancer patients (HR = 1.714, 95% CI: 1.134–2.590, *p* = 0.011; Table 4). In adjustment for age, gender, smoking, betel quid chewing, and N value, EIF5A2 expression was still statistically significant with the overall survival (HR = 1.520, 95% CI: 1.002–2.307, *p* = 0.049; Table 5).

## 4. Discussion

EIF5A is a small, abundant, highly-conserved RNA-binding protein that is involved in the human cell cycle, apoptosis, and viral replication [28]. This translation factor family is well known for the unique protein hypusine, which underlines their importance in interfering with ribosome, cytoskeleton, and nuclear-cytoplasmic transportation [11]. Both isoforms of EIF5A have been investigated for their association with human cancer malignances, while EIF5A2 has been shown to be involved in tumor progression, poor prognosis, and lymph node metastasis [16,29,30]. Overexpression of EIF5A2 has been observed in several cancers with less favorable clinical features and outcomes. Although EIF5A2 has not become an invariable hallmark of cancer due to its poor expression in some lymphoma and leukemia cell lines, there is a lack of studies on the relationship between EIF5A2 expression and oral cancer [31].

While oral cancer is said to be easily diagnosed at an early stage, its five-year survival rate still has not improved over recent years due to tumor recurrences and metastasis [32]. While more prognostic biomarkers for oral cancer are needed for early stages, limited evidence for EIF5A2 been reported. In this study, we analyzed multiple clinical parameters of 272 oral cancer patients along with EIF5A2 expression, providing evidence indicating that EIF5A2 has a potential role in oral cancer clinical prognosis. Our results for the oral cancer patient sample studied with IHC staining demonstrated that an increase in EIF5A2 expression is significantly related to poor overall survival rate as patients with regional lymph node metastasis showed higher EIF5A2 expression. Moreover, as analyzed by univariate and multivariate analyses, EIF5A2 is potentially an independent prognostic biomarker unaffected by gender, smoking, or quid chewing. This is consistent with the results obtained by a previous study showing that the upregulation of EIF5A2 during oral squamous cell carcinoma progression and lymph node metastasis predicted EIF5A2 as a possible new biomarker [33].

Further studies of EIF5A2 associated with tumor metastasis have indicated EMT as the major pathway. EMT is an essential reversible mechanism of tissue repair and embryonic development that also contributes to cancer progression [34]. In colorectal cancer, the overexpression of EIF5A2 has been reported to promote cancer aggressiveness by upregulating MTA1 to induce EMT [21]. Another colorectal cancer study has demonstrated that EIF5A2 plays an important role in the chemoresistance to doxorubicin through EMT regulation [30]. The knockdown of EIF5A2 in bladder cancer suggests that by increasing the enrichment of STAT3, EIF5A2 elevated TGF TGFβ1 expression by inducing EMT [17]. Increased expression of EIF5A2 has been suggested to enhance melanoma cell invasion with increasing MMP-2. The same study also found the upregulation of vimentin, fibronectin, and *α*-SMA and downregulation of E-cadherin in EIF5A2-overexpressing melanoma cells, suggesting that EIF5A2 might induce EMT [35]. On the other hand, EMT in oral squamous cancer cells has been shown to be mediated by multiple growth factors and signaling pathways, including EGF, TGFβ1, P13k/Akt, MEK/ERK, and Wnt/β-catenin pathways and associated with poor prognosis [32,36]. Therefore, with a notable correlation between EIF5A2 expression and oral cancer regional lymph node metastasis, we speculate that overexpression of EIF5A2 may result in an advanced stage of oral cancer by inducing EMT. Based on our results, we can imply that EIF5A2 expression effects overall survival rates through the EMT pathway. These evidences supported the clinical application of N1-guanyl-1,7-diaminoheptane (GC7, a novel eIF5A2 inhibitor) in OSCC [37]. The combination of GC7 and cisplatin reversed the upregulation of p-STAT3, c-Myc, and cisplatin-promoted mesenchymal–epithelial transition and, further, contributed in significant tumor volume reduction without distinct body weight loss in animal model [37]. The combination therapy may offer an efficient and safe therapeutic alternative for OSCC patients.

There are some limitations to this study. First, the sample size is limited by both case number and region, central Taiwan. Thus, a larger sample size is needed to strengthen the impact of our findings. Second, tissue staining via tissue arrays instead of whole mount staining cannot represent whole tumor condition and provide only data on tissue cores. In addition, the interobserver variability was not assessed. Third, relapse-free survival and disease-free survival was not investigated, and only overall survival was considered in this study. Adjuvant therapies were not investigated. Thus, more complete studies and in-depth experiments are required in the future.

In conclusion, our findings have demonstrated that the increased expression of EIF5A2 in oral cancer patients is associated with regional lymph node metastasis and poor overall survival rate. Further, our study is the first to support that EIF5A2 may result in tumor malignancy via the EMT pathway, indicating EIF5A2 as a possible prognostic biomarker, which has important clinical significance for judging the clinical prognosis of oral cancer.

## Figures and Tables

**Table 1:** Relationships between EIF5A2 expression and clinical parameters in oral cancer patients.

		EIF5A2 Expression		
Parameters	Case Number	Low	High	*p*-Value
Age (year)		56.1±12.4	56.8±11.0	0.655
Gender				
Female	42	15 (35.7)	27 (64.3)	0.878
Male	230	85 (37.0)	145 (63.0)	
Smoking				
No	160	55 (34.4)	105 (65.6)	0.329
Yes	112	45 (40.2)	67 (59.8)	
Betel quid chewing				
No	220	83 (37.7)	137 (62.3)	0.498
Yes	52	17 (32.7)	35 (67.3)	
Differentiation				
Well	42	14 (33.3)	28 (66.7)	0.616
Moderate + Poor	230	86 (37.4)	144 (62.6)	
Stage				
I	55	22 (40.0)	33 (60.0)	0.577
II + III + IV	217	78 (35.9)	139 (64.1)	
T value				
1	71	26 (36.6)	45 (63.4)	0.976
2 + 3 + 4	201	74 (36.8)	127 (63.2)	
N value				
0	168	72 (42.9)	96 (57.1)	0.008
1 + 2 + 3	104	28 (26.9)	76 (73.1)	

**Table 2:** Relationships between EIF5A2 expression and epithelial–mesenchymal transition markers in oral cancer patients.

		EIF5A2 Expression		
Parameters	Case Number	Low	High	*p*-Value
E-cadherin ^1^				
Low	120	34 (28.3)	86 (71.7)	0.046
High	142	57 (40.1)	85 (59.9)	
Beta-catenin ^2^				
Low	135	58 (43.0)	77 (57.0)	0.020
High	133	39 (29.3)	94 (70.7)	

^1^ Missing data due to IHC staining detachment: *n* = 10. ^2^ Missing data due to IHC staining detachment: *n* = 4.

**Table 3:** Univariate analysis of the influence of various parameters on the overall survival of oral cancer patients.

		Overall Survival			
Parameter	Category	5-year Survival (%)	HR	95% CI	*p*-Value
Age	≥57/<57	60.9/61.0	1.061	0.749–1.503	0.739
Gender	Male/Female	59.7/71.4	1.477	0.862–2.531	0.156
Smoking	Yes/No	60.4/62.1	1.017	0.716–1.445	0.924
Betel quid chewing	Yes/No	62.6/60.9	0.851	0.533–1.359	0.500
Stage	II+III+IV/I	57.9/74.4	1.762	1.082–2.868	0.023
N value	1+2+3/0	36.8/68.4	2.770	1.906–4.024	<0.001
EIF5A2	High/Low	49.7/67.3	1.696	1.126–2.554	0.011

**Table 4:** Multivariate analysis of the influence of various parameters on the overall survival of oral cancer patients.

		Overall Survival			
Parameter	Category	Mean Survival (years)	HR *	95% CI	*p*-Value
Age	≥57/<57	4.8/4.9	0.837	0.570–1.230	0.365
Gender	Male/Female	4.8/5.5	1.347	0.736–2.468	0.334
Smoking	Yes/No	4.9/4.9	0.805	0.510–1.271	0.351
Betel quid chewing	Yes/No	4.8/5.1	0.750	0.416–1.352	0.338
Stage	II+III+IV/I	4.7/5.6	1.950	1.123–3.387	0.018
EIF5A2	High/Low	4.3/5.2	1.714	1.134–2.590	0.011

* Adjusted for age, gender, smoking, betel quid, and stage.

**Table 5:** Multivariate analysis of the influence of various parameters on the overall survival of oral cancer patients.

		Overall Survival			
Parameter	Category	Mean Survival (yrs)	HR *	95% CI	*p*-Value
Age	≥57/<57	4.8/4.9	0.913	0.622–1.339	0.640
Gender	Male/Female	4.8/5.5	1.704	0.931–3.119	0.084
Smoking	Yes/No	4.9/4.9	0.809	0.514–1.274	0.361
Betel quid chewing	Yes/No	4.8/5.1	0.779	0.434–1.397	0.402
N value	1+2+3/0	3.4/5.4	2.699	1.847–3.943	<0.001
EIF5A2	High/Low	4.3/5.2	1.520	1.002–2.307	0.049

* Adjusted for age, gender, smoking, betel quid, and N value.
